# Decreased pain threshold and enhanced synaptic transmission in the anterior cingulate cortex of experimental hypothyroidism mice

**DOI:** 10.1186/1744-8069-10-38

**Published:** 2014-06-18

**Authors:** Jun Yi, Jian-yong Zheng, Wei Zhang, Shan Wang, Zhi-fu Yang, Ke-feng Dou

**Affiliations:** 1 Department of General Surgery, Xijing Hospital; The Fourth Military Medical University, Xi’an 710032, China; 2 State Key Laboratory of Cancer Biology and Institute of Digestive Diseases, Xijing Hospital; The Fourth Military Medical University, Xi’an 710032, China; 3 Department of Pharmacy, Xijing Hospital; The Fourth Military Medical University, Xi’an 710032, China

**Keywords:** Thyroid hormone, Pain, Anterior cingulate cortex

## Abstract

**Background:**

Thyroid hormones are essential for the maturation and functions of the central nervous system. Pain sensitivity is related to the thyroid status. However, information on how thyroid hormones affect pain processing and synaptic transmission in the anterior cingulate cortex (ACC) is limited. Nociceptive threshold and synaptic transmission in the ACC were detected in the experimental hypothyroidism (HT) mice.

**Results:**

HT was induced by methimazole and potassium perchlorate in distilled drinking water for 4 weeks. The threshold of pain perception to hot insults, but not mechanical ones, decreased in hypothyroid mice. After treatment with tri-iodothyronine (T3) or thyroxine (T4) for 2 weeks, thermal pain threshold recovered. Electrophysiological recordings revealed enhanced glutamatergic synaptic transmission and reduced GABAergic synaptic transmission in the ACC. Supplementation with T3 or T4 significantly rescued this synaptic transmission imbalance. In the same model, HT caused the up-regulation of the GluR1 subunit of the α-amino-3-hydroxy-5-methyl-4-isoxazolepropionic acid receptor and NR2B-containing *N*-methyl-D-aspartate receptors, but it down-regulated γ-aminobutyric acid A receptors in the ACC. Supplementation with T3 or T4 notably recovered the levels of above proteins.

**Conclusions:**

These results suggest that HT promotes hypersensitivity to noxious thermal, and that supplementation with T3 or T4 rescues the imbalance between excitatory and inhibitory transmission in the ACC.

## Background

The thyroid hormones tri-iodothyronine (T3) and thyroxine (T4) are essential for the metabolic homeostasis of the tissues. Moreover, these hormones are crucially involved in the maturation and function of the central nervous system [1]. Congenital hypothyroidism (HT) causes mental retardation in children [2]. Adult onset of HT also decreases cognition and increases anxiety [3]. The pain sensitivity has been documented to be related with thyroid status [4]. An adequate support of maternal thyroid hormones may be required to ensure a normal nociceptive function of offspring into adulthood [5]. However, information regarding the underlying mechanism of thyroid hormones in affecting pain processing and synaptic transmission in the anterior cingulate cortex (ACC), which is highly involved in the pain modulation, is limited.

ACC is a critical brain region involved in learning, memory, attention, and pain [6-9]. Recent studies using different experimental approaches suggest that ACC has important functions in processing pain-related information in humans and in behavioral responses to noxious stimuli or tissue injury in animals [10-12]. Moreover, an ample group of neurotransmitters and chemical messengers, including opioids, glutamate, GABA, and dopamine, are involved in the modulation of pain by these cortical structures. 3-iodothyronamine (T1AM) is an endogenous derivative of thyroid hormones and is a rapid modulator of behavior and metabolism [4]. Electrophysiological experiments suggest that T1AM affects the response to catecholamines and other neurotransmitters by functioning as a specific inhibitor of dopamine, noradrenaline re-uptake, and monoamine transport into synaptic vesicles [13]. Therefore, T1AM might be regarded as a neuromodulator.

The balance between excitatory and inhibitory transmission is critical for brain functions. The prolonged disturbance of this balance can promote pathological anxiety-like behaviors [14] and nociceptive responses [15]. Enhanced excitatory and reduced inhibitory synaptic transmissions contribute to persistent pain-induced neuronal hyper-responsiveness in the ACC [16]. Studies have often demonstrated the function of the ACC in the modulation of anticipatory nociceptive responses aimed at avoidance of potential danger, without changing nociceptive motor responses [17], and the modulation of responses to aversive emotional content, which demonstrates its broad function in many types of emotional processing [18]. In present study, we found that drugs-induced HT promoted hypersensitivity to noxious thermal but not mechanical stimulus, and that supplementation with T3 or T4 reversed the imbalance between excitatory and inhibitory transmission in ACC.

## Results

### T3 and T4 levels in the serum

**Figure 1:**
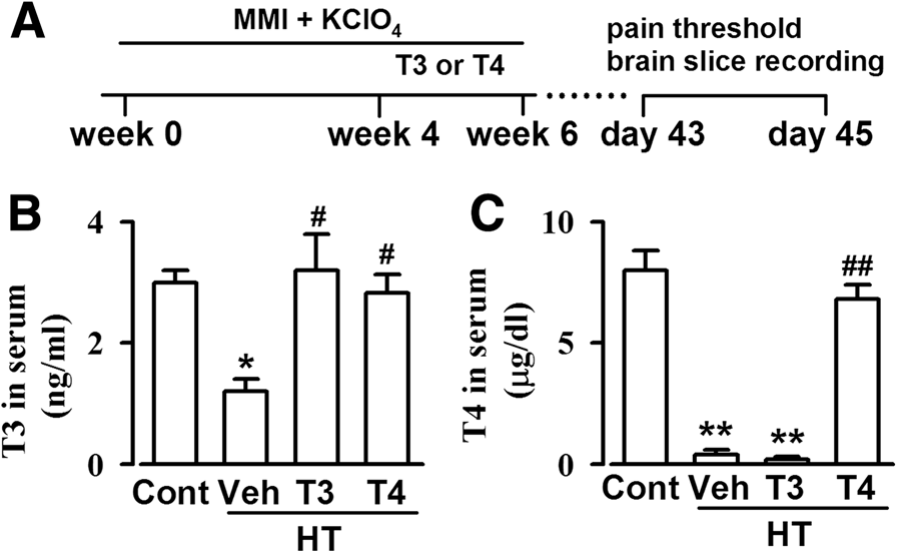
**Levels of T3 and T4 in serum. (A)** Timeline for experiment. Mice were treatment with MMI and KClO_4_ for 4 weeks, then followed with T3 or T4 supplementation for 2 weeks. Detections were performed during Day 43 and Day 45. Levels of T3 **(B)** and T4 **(C)** in saline control, vehicle-, T3-, and T4-treated HT mice. n = 5 in each group. * *p* < 0.05, ** *p* < 0.01 compared with naïve saline control; ^##^*p* < 0.01 compared with vehicle-treated HT mice.

Methimazole (MMI) and 1% potassium perchlorate (KClO_4_) treatment for 4 weeks significantly reduced the serum free levels of T3 and T4 (Figure 1B and C). Supplementation with T3 significantly increased the serum levels of T3, but not T4. However, supplementation with T4 significantly increase the serum levels of T3 and T4 in the MMI and KClO_4_ treated mice.

### Pain threshold in the male hypothyroid mice

**Figure 2:**
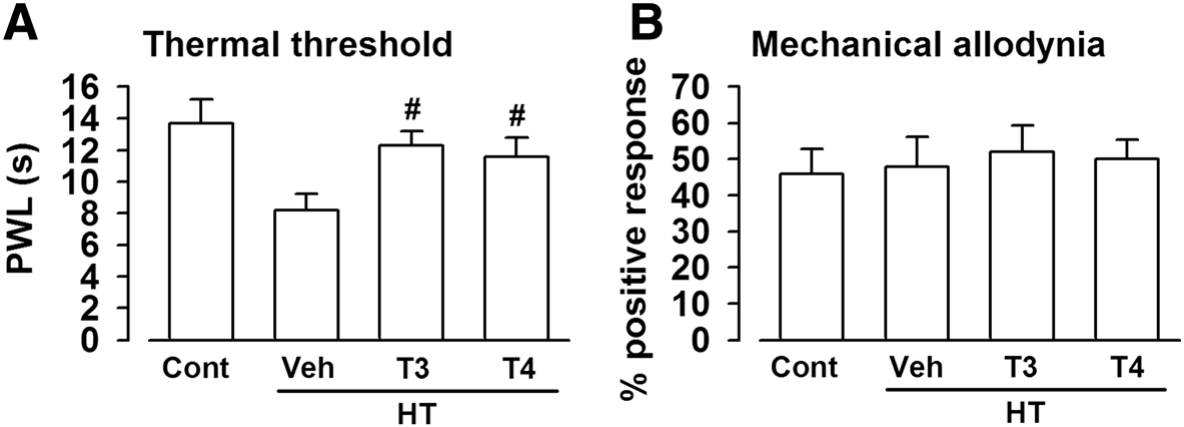
**Decreased pain threshold in hypothyroid mice. (A)** Thermal threshold was shown as PWL in saline control, vehicle-, T3-, and T4-treated HT mice. **(B)** Mechanical pain threshold in saline control, vehicle-, T3-, and T4-treated HT mice. n = 5 in each group. ^#^*p* < 0.05 compared with vehicle-treated HT mice.

The thyroid hormone receptors were highly expressed in the brain. First, we investigated the effect of experimental HT on the nociceptive threshold. Paw withdrawal latency (PWL) was detected by investigating the latency to licking of mice. MMI and KClO_4_ treatment for 4 weeks significantly reduced the threshold of pain perception to hot insults (Figure 2A). The succeeding treatment with T3 or T4 for 2 weeks recovered the paw-withdraw latency in the hypothyroid mice (Figure 2A). However, mechanical pain threshold did not differ in the hypothyroid mouse model and supplementation with T3 or T4 (Figure 2B). These data indicate that thermal pain sensitivity is related to the thyroid status but not to mechanical pain.

### Enhanced glutamatergic transmission in the ACC of hypothyroid mice

**Figure 3:**
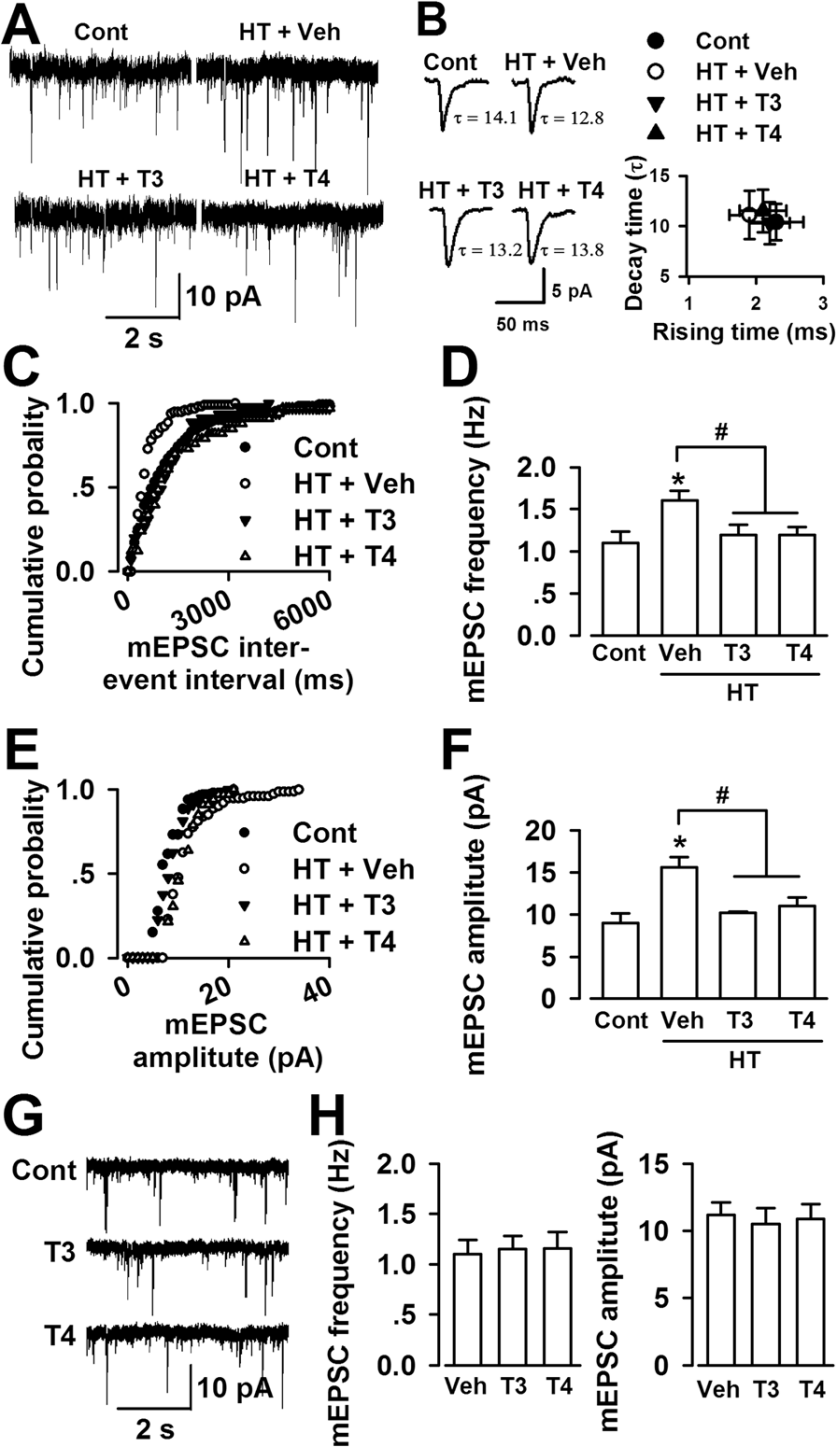
**Enhanced glutamatergic synaptic transmission in the ACC. (A)** Sample mEPSCs recording in pyramidal neurons at a holding potential of -70 mV from each group. **(B)** Left, average mEPSC of events from saline (52 events), vehicle- (67 events), T3- (61 events), and T4- (57 events) treated HT mice; Right, Time constant of mEPSC decay (τ) versus the rising time (10 –90%) in the recordings from saline control (n = 10 slices/5 mice), vehicle- (n = 9 slices/5 mice), T3- (n = 9 slices/5 mice), and T4- (n = 8 slices/5 mice) treated HT mice. **(C)** Cumulative frequency histogram of the mEPSCs from the slices in each group. **(D)** Summery of mEPSCs frequency in neurons from saline control, vehicle-, T3-, and T4-treated HT mice. **(E)** Cumulative amplitude histogram of the mEPSCs from the slices in each group. **(F)** Summery of mEPSCs amplitude in neurons from control, vehicle-, T3-, and T4-treated HT mice. (D) and (F): Number of recordings from control (n = 11 neurons/5 mice), vehicle- (n = 10 neurons/5 mice), T3- (n = 9 neurons/5 mice), and T4- (n = 8 neurons/5 mice) treated HT mice. * *p* < 0.05 compared with saline control; ^#^*p* < 0.05 compared with vehicle-treated HT mice. **(G)** Sample mEPSCs recording in pyramidal neurons at a holding potential of -70 mV from naïve mice. **(H)** Summery of mEPSCs frequency (left) amplitude (right) in neurons perfused with saline (n = 8 neurons/4 mice), T3 (n = 10 neurons/4 mice), and T4 (n = 8 neurons/4 mice).

The ACC is involved in pain processing [6,9]. To examine the function of the thyroid hormone in the balance between excitation and inhibition, we performed whole-cell patch-clamp recordings to record synaptic transmission in the ACC pyramidal neurons. Treatment with MMI and KClO_4_ induced the increase of glutamatergic transmission in the ACC as shown by the increase of frequency (Figure 3C and D) or amplitude of mEPSCs (Figure 3E and F). Supplementation with T3 or T4 significantly reduced the frequency and amplitude of mEPSCs in the ACC pyramidal neurons of the hypothyroid mice (Figure 3D and F). The kinetics of mEPSCs were not altered among the neurons from the saline control, vehicle-, T3-, and T4-treated hypothyroid mice (Figure 3B). However, bath perfusion of T3 (4 nM) or T4 (40 nM) did not alter the frequency and amplitude of mEPSCs in the ACC pyramidal neurons of the naïve mice (Figures 3G and H). Increase in the mEPSCs frequency suggests the enhanced presynaptic release probability in the hypothyroid mice.

### Decreased inhibitory synaptic transmission in the ACC of hypothyroid mice

**Figure 4:**
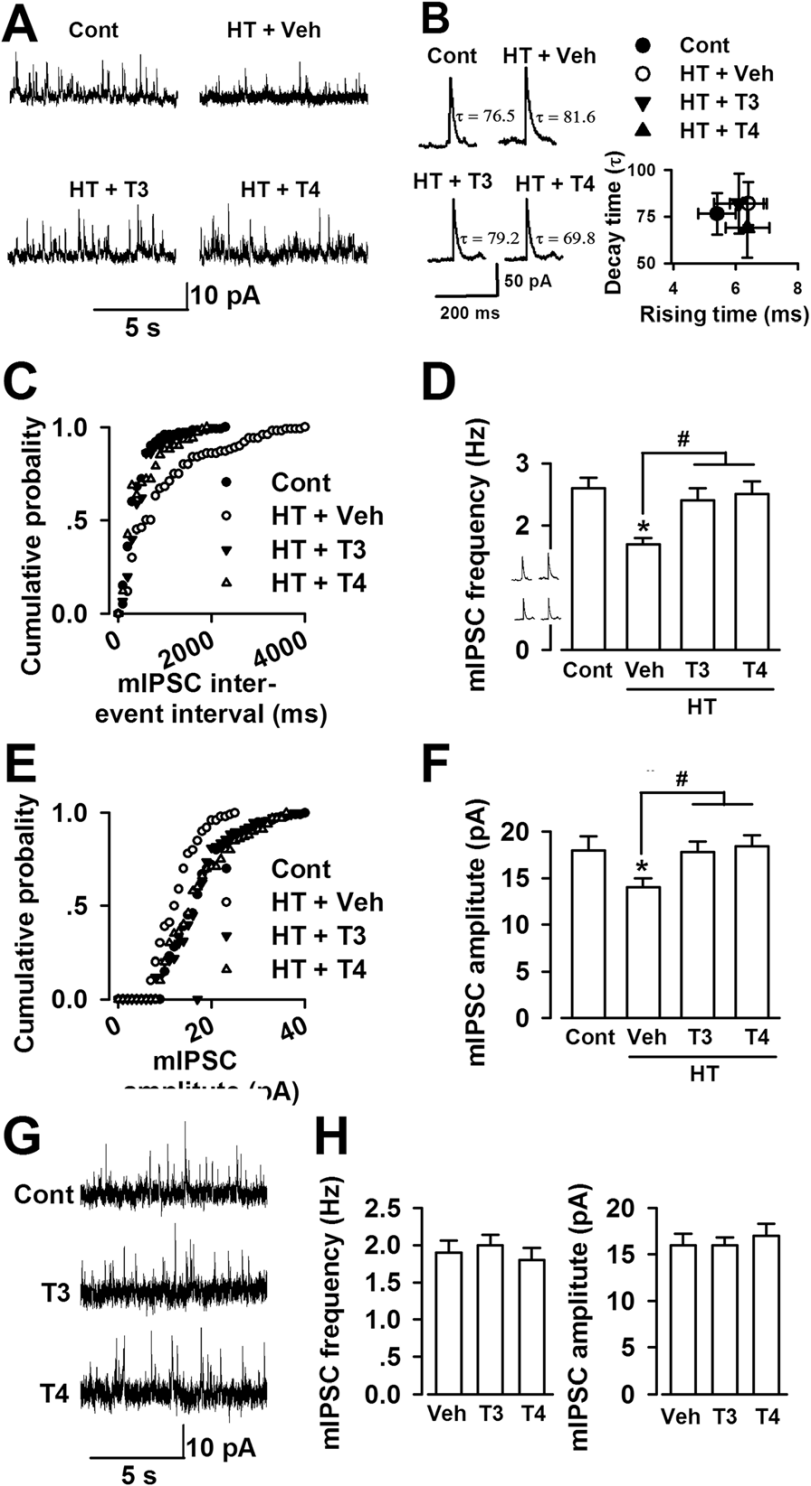
**Reduced GABAergic synaptic transmission in the ACC. (A)** Sample mIPSCs recording in pyramidal neurons at a holding potential of 0 mV from each group. **(B)** Left, average mIPSC of events from saline (89 events), vehicle- (97 events), T3- (82 events), and T4- (94 events) treated HT mice; Right, Time constant of mIPSC decay (τ) versus the rising time (10 –90%) in the recordings from saline control (n = 8 slices/5 mice), vehicle- (n = 8 slices/5 mice), T3- (n = 9 slices/5 mice), and T4- (n = 10 slices/5 mice) treated HT HT mice. **(C)** Cumulative frequency histogram of the mIPSCs from the slices in each group. **(D)** Summery of mIPSCs frequency in neurons from saline control, vehicle-, T3-, and T4-treated HT mice. **(E)** Cumulative amplitude histogram of the mIPSCs from the slices in each group. **(F)** Summery of mIPSCs amplitude in neurons from saline control, vehicle-, T3-, and T4-treated HT mice. **(D)** and **(F)**: Number of recordings from control (n = 9 neurons/5 mice), vehicle- (n = 11 neurons/5 mice), T3- (n = 10 neurons/5 mice), and T4- (n = 9 neurons/5 mice) treated HT mice. * *p* < 0.05 compared with control; ^#^*p* < 0.05 compared with vehicle treated-HT mice. **(G)** Sample mIPSCs recording in pyramidal neurons at a holding potential of 0 mV from naïve mice. **(H)** Summery of mIPSCs frequency (left) amplitude (right) in neurons perfused with saline (n = 8 neurons/4 mice), T3 (n = 9 neurons/4 mice), and T4 (n = 9 neurons/4 mice).

To further examine the function of thyroid hormone in the balance between excitation and inhibition, we recorded mIPSCs in the ACC pyramidal neurons. Treatment with MMI and KClO_4_ induced the decrease of GABAergic transmission in the ACC, as demonstrated by the decrease in frequency (Figure 4A, and C–D) or amplitude of mIPSCs (Figure 4E and F). Supplementation with T3 or T4 significantly induced the frequency and amplitude of mIPSCs in the ACC pyramidal neurons from hypothyroid mice (Figure 4D and F). The kinetics of mIPSCs were not altered among the neurons from the saline control, vehicle-, T3-, and T4-treated hypothyroid mice (Figure 4B). Similar to the effects on mEPSCs, bath perfusion of T3 (4 nM) or T4 (40 nM) did not alter the frequency and amplitude of mIPSCs in the ACC pyramidal neurons of the naïve mice (Figure 4G and H).

### Supplementation with thyroid hormone reverses glutamate receptor up-regulation

**Figure 5:**
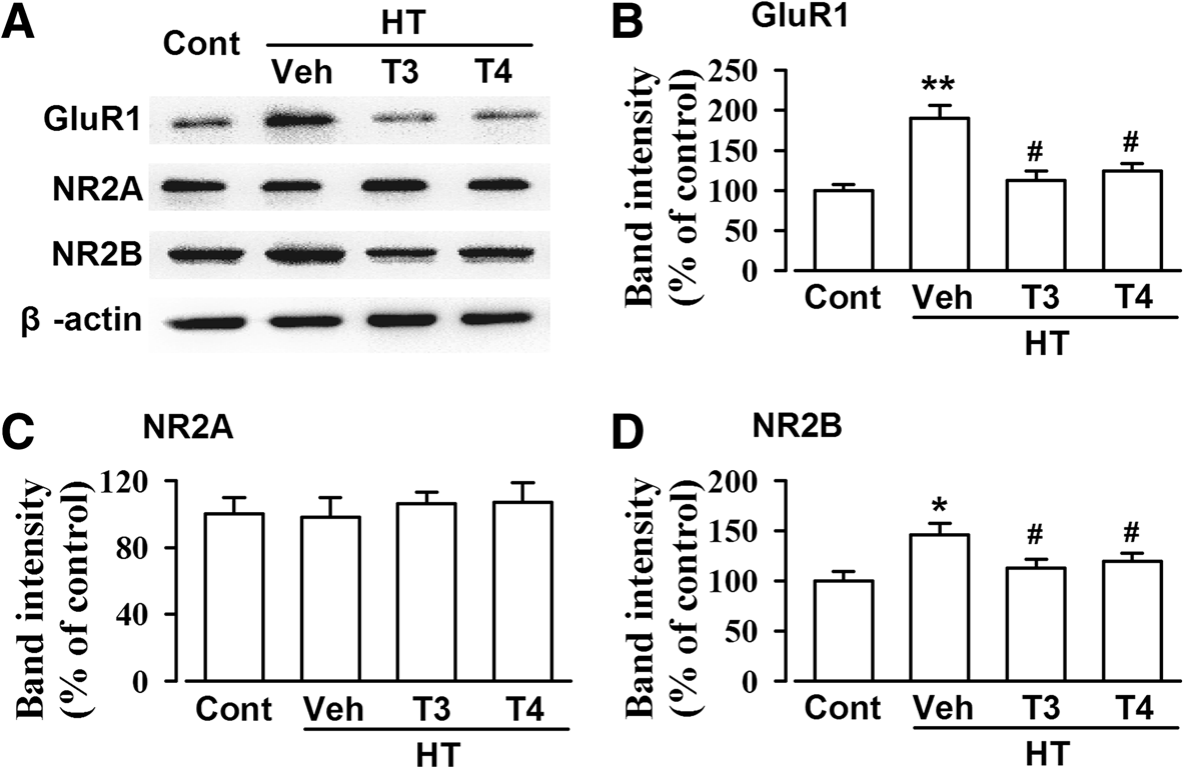
**Expression of glutamate receptors. (A)** Representative western-blot analysis of GluR1, NR2A, and NR2B subunits in the ACC homogenates. Levels of GluR1 **(B)**, NR2A **(C),** and NR2B **(D)** in saline control, vehicle-, T3-, and T4-treated HT mice. n = 5 in each group. * *p* < 0.05, ** *p* < 0.01 compared with saline control; ^#^*p* < 0.05 compared with vehicle-treated HT mice.

To further determine the contribution of postsynaptic component to the enhanced glutamatergic transmission in the ACC of the hypothyroid mice, we detected the expression of glutamate receptors by Western blot (Figure 5A). Treatment with MMI and KClO_4_ induced the up-regulation of GluR1 (Figure 5B) and NR2B (Figure 5D) subunits in the ACC. Supplementation with T3 or T4 reversed GluR1 and NR2B up-regulation (Figures 5B and D). However, NR2A subunit was not altered in the ACC from hypothyroid mice and T3/T4 supplemented hypothyroid mice (Figure 5D).

### Supplementation with thyroid hormone reverses GABA_A_ receptor down-regulation

**Figure 6:**
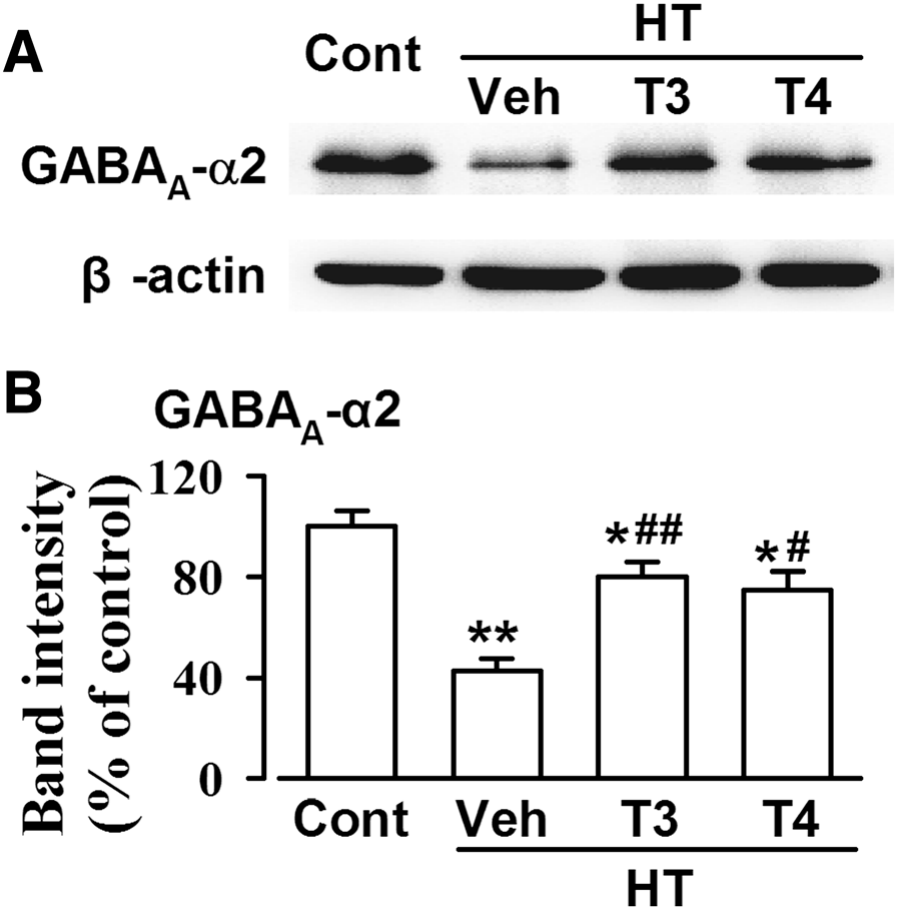
**Expression of GABA**_**A **_**receptor. (A)** Representative western-blot analysis of GABA_A_-α2 subunit in the ACC homogenates. **(B)** Levels of GABA_A_-α2 in saline control, vehicle-, T3-, and T4-treated HT mice. n = 5 in each group. * *p* < 0.05, ** *p* < 0.01 compared with saline control; ^#^*p* < 0.05, ^##^*p* < 0.01 compared with vehicle-treated HT mice.

GABA_A_ receptors are composed of α, β, and γ subunits. GABA_A_ receptor containing α2 subunit is highly represented in frontal cortex [19]. Treatment with MMI and KClO_4_ induced a significant decrease in the expression of GABA_A_-α2 in the ACC (Figure 6A and B). Supplementation with T3 or T4 reversed the GABA_A_-α2 down-regulation (Figure 6B). These results demonstrate that the replacement of thyroid hormones reverses the down-regulation of GABA_A_ receptor expression in the hypothyroid mice.

## Discussion

This study provides strong evidence for the function of thyroid hormones in pain processing. Adult onset of HT promotes hypersensitivity to noxious thermal but not mechanical stimulus. The hypersensitivity to noxious thermal in HT is relieved by T3 or T4 replacement. The underlying mechanisms of hormone therapy are presumed to maintain a balance between glutamatergic and GABAergic transmission in the ACC of hypothyroid mice.

### HT and pain

In vertebrates, the thyroid secretes predominantly T4, which has to be converted into the active hormone T3 and is responsible for most of the T3 supply within the brain [20]. T3 and T4 bind three members of the nuclear receptor superfamily: TRα1, TRβ1, and TRβ2 [21]. These receptors regulate gene transcription by both ligand-dependent and ligand-independent means [22]. The thyroid hormone receptors are highly expressed in the brain and have critical roles in brain development and high functions [1,2]. Pain sensitivity is related to the thyroid status [4]. The present study found that HT increases hypersensitivity to noxious thermal but not mechanical stimulus. The hot-plate thermal model could be interpreted as a behavioral reaction to emotional content, which is produced in a state of acute pain and in a scenario wherein the animal cannot escape from the source of stimulation. Studies have shown the function of the ACC in the modulating nociceptive responses aimed at avoiding potential danger [17]. ACC has also been implicated in the modulation of responses to aversive emotional processing [15]. HT produces depression and anxiety in rodent and patients [23,24]. This result may partially explain the hypersensitivity to noxious thermal stimulus in the hypothyroid mice.

### Thyroid hormones, ACC synaptic transmission and plasticity

ACC is a critical brain region involved in processing pain-related information in humans and in behavioral responses to noxious stimuli or tissue injury in animals [10-12]. In the present study, we found that synaptic transmission was altered in the ACC synapses in the hypothyroid mice. We also established that the replacement of thyroid hormone could reverse the synaptic alterations. The adult onset of HT prevents the generation of experimentally evoked early and long-term potentiation in the hippocampal CA1 region of the rat, which is associated by the changes in the expression of NR1 glutamatergic receptor subunits [25,26]. These findings indicate that thyroid hormones affect nociceptive responses, at least partially, through regulating the synaptic transmission in the ACC. Previous studies have showed the increased quantal release of excitatory transmitter in anterior cingulate cortex of adult mice with chronic pain [27,28]. Our present findings are consistent with these studies. Furthermore, we found that the acute perfusion of T3 or T4 in the brain slices did not induce the alterations in the frequency and amplitude of mEPSCs and mIPSCs. As mentioned above, thyroid hormone receptors are nuclear receptors and regulate gene transcription by both ligand-dependent and ligand-independent means [22]. Thyroid hormone replacement therapy occurs through gene transcription and protein expression.

### Thyroid hormones and the balance between excitatory and inhibitory transmission

Excitatory and inhibitory transmission homeostasis is critical for brain functions [14,15]. Enhanced excitatory and reduced inhibitory synaptic transmissions contribute to persistent pain-induced neuronal hyper-responsiveness in the ACC [16]. Systemic or ACC local administration of Ro25-6981, which is a specific NR2B receptors antagonist, significantly reduces behavioral allodynia [11]. In the present study, we found that HT induced the up-regulation of GluR1 and NR2B-containing NMDA receptors and the down-regulation of α2-containing GABA_A_ receptors in the ACC. GABA_A_ receptors consist of α, β, and γ subunits. GABA_A_ receptors containing α1-, α2-, β1-3, and γ2-subunits are highly represented in frontal cortex [29]. α2-containing GABA(A) receptor, representing approximately 15-20% of all GABA_A_ receptors, is considered as a target for the development of novel treatment strategies for CNS disorders, such as anxiety, depression, schizophrenia, and chronic pain. Western blot data indicated that the replacement of T3 or T4 reversed the levels of GluR1, NR2B-containing NMDA receptors and α2-containing GABA_A_ receptors in the hypothyroid mice. Combining these data with electrophysiological recordings, we could further confirm the glutamatergic and GABAergic synaptic transmission changes induced by thyroid hormones. We did not exclude the possible alterations of other GABA_A_ subunits in the hypothyroid mice.

ACC is well known for its critical in the pain processing, including the chronic inflammatory pain, neuropathic pain, and the phantom limb pain [28,30-33]. In the present study, we found the parallel changes of nociceptive behavior and electrophysiology in HT mice and rescued by T3 and T4. Furthermore, the changes in E/I imbalance was found in the HT mice. Actually, enhancement of GABAergic transmission is a useful therapeutic strategy for the pain relief [34,35]. Thus, restoring the E/I imbalance in ACC may contribute to decrease the hypersensitivity after supplementation with T3 or T4. Indeed, we could exclude the possible changes of E/I imbalance in other pain-related brain regions of HT mice, such as insular cortex, amygdala, and somatosensory cortex.

## Conclusions

In summary, this study investigated the novel mechanisms underlying thyroid status and nociceptive processing. Thyroid hormone replacement is associated with restoring the balance between excitatory and inhibitory transmission in the ACC of HT. This phenomenon indicates that a signal transduction mechanism is necessary for neuronal function under both physiological and pathological conditions. Accordingly, improved understanding of the effect of thyroid status on pain control is beneficial.

## Methods

### Materials

All chemicals and reagents were purchased from Sigma (St. Louis, MO) unless otherwise stated. Rabbit anti-NR2A and anti-NR2B were purchased from Millipore (Billerica, MA). Rabbit anti-GluR1 was purchased from Abcam (Cambridge, UK). Rabbit anti-GABA_A_-α2 antibody was purchased from Alomone labs (Jerusalem, Israel). All of the chemicals and reagents used were commercially available and of standard biochemical quality.

### Animals and treatment

Male C57BL mice, aged 7 to 8 weeks obtained from the Laboratory Animal Center of the Fourth Military Medical University (FMMU), were used in all the experiments. Animals were housed in groups of 4, with food and water available *ad libitum*. The room was maintained at controlled temperature (24 ± 2°C), humidity (50 – 60%). Mice were allowed to adapt to laboratory conditions for at least 1 week before procedures were performed. All animal experiments were carried out in accordance with the National Institutes of Health Guide for the Care and Use of Laboratory Animals (NIH Publications No. 80–23, revised 1978) to minimize animal suffering. The Fourth Military Medical University Animal Care and Use Committee approved the animal protocols. To established hypothyroidism, mice were administered with 0.05% methimazole (MMI) and 1% potassium perchlorate (KClO_4_) in distilled drinking water for 6 weeks [24]. From week 4, some of mice were supplemented with T3 (0.5 μg/ml) or T4 (5 μg/ml) for 2 weeks. T3 and T4 hormones were added in drinking water. Behavioral testing, electrophysiological recording, and western-blot were performed between Day 43 and Day 45 (Figure 1A).

### Thermal threshold

The hot-plate test was used to measure the response latencies in mice according to the previously described method [36]. The mice were screened by placing them on a hot metal plate maintained at 50 ± 0.05°C. The paw withdrawal latency (PWL) was defined as the time to start jumping, withdrawal of the paws or the licking of the paws. The mice were selected for the experiment which offered response within 20 s. The cut-off time was set at 30 s to minimize skin damage.

### Mechanical threshold

Mice were placed in individual plastic boxes and allowed to adjust to the environment for 30 min. Using the up-down paradigm [37], mechanical sensitivity was assessed with a set of von Frey filaments (0.04–15 g) applied perpendicularly to the midplantar surface of the hind paws. Based on preliminary experiments that characterized the threshold stimulus, the innocuous 0.4 g (#3.61) filament, representing 50% of the threshold force, was used to detect mechanical threshold. The filament was applied to the point of bending six times each to the dorsal surfaces of the injected hind paws. Positive responses included prolonged hind-paw withdrawal followed by licking or scratching. For each time point, the percentage response frequency of hind-paw withdrawal was expressed as follows: (number of positive responses)/6 × 100 per hind-paw.

### Thyroid measurements

T3 and T4 levels in the plasma were detected using the ELISA kit (IBL, MN) following the manufacturer’s instructions. Briefly, the mice were anesthetized with diethyl ether. The eyeballs of mice were removed by ophthalmological forceps to break the blood vessels of the fundus oculi. Blood (about 1.0 mL every mouse) was collected in the 2.0 ml centrifuge tube. Blood was centrifuged at 12000 rpm at 4°C for 15 min and the supernatant was collected for ELISA.

### Electrophysiological recordings

Coronal brain slices (300 μm), containing the ACC, were prepared as previously described [9]. Slices were transferred to submerged recovery chamber with oxygenated (95% O_2_ and 5% CO_2_) artificial cerebrospinal fluid (ACSF) containing (in mM): 124 NaCl, 2.5 KCl, 2 CaCl_2_, 2 MgSO_4_, 25 NaHCO_3_, 1 NaH_2_PO_4_, 10 glucose at room temperature for at least 1 h. Experiments were performed in a recording chamber on the stage of an Axioskop 2FS microscope with infrared DIC optics for visualization of whole-cell patch clamp recording. Miniature postsynaptic currents (mEPSCs and mIPSCs) were recorded from layer II–III neurons with an Axon 200B amplifier (Axon Instruments, CA) with 0.5 μM TTX in the ACSF. AMPA receptor mediated mEPSCs were recorded in the neurons clamped at -70 mV. The recording pipettes (3–5 MΩ)? were filled with solution containing (mM) 145 K-gluconate, 5 NaCl, 1 MgCl_2_, 0.2 EGTA, 10 HEPES, 2 Mg-ATP, and 0.1 Na_3_-GTP (adjusted to pH 7.2 with KOH). GABA_A_ receptor-mediated mIPSCs were recorded in the neurons clamped at 0 mV with CNQX (20 μM) and AP-5 (100 μM) in the ACSF. The patch electrodes contained 102 mM cesium gluconate, 5 mM TEAchloride, 3.7 mM NaCl, 11 mM BAPTA, 0.2 mM EGTA, 20 mM HEPES, 2 mM MgATP, 0.3 mM NaGTP, and 5 mM QX-314 chloride (adjusted to pH 7.2 with CsOH). Access resistance was 15–30 MΩ? and monitored throughout the experiment. Data were discarded if access resistance changed more than 15% during an experiment.

### Western-blot analysis

Western-blot analysis was performed as previously described [38]. Tissue samples from the ACC were dissected from brain slices (300 μm) under an anatomical microscope. ACC samples from 5 mice were analyzed by Western-blot. Equal amounts of protein (50 μg) were separated and electrotransferred onto PDVF membranes (Invitrogen), which were probed with antibodies against GluR1 (dilution ratio 1:300), NR2A (dilution ratio 1:200), NR2B (dilution ratio 1:500), and GABA_A_-α2 (dilution ratio 1:200) with β-actin (dilution ratio 1:10000) as the loading control. The membranes were incubated with horseradish peroxidase–conjugated secondary antibodies (anti-rabbit/anti-mouse IgG for the primary antibodies). The densitometric analysis of the Western-blot was conducted using a ChemiDoc XRS (Bio-Rad, Hercules, CA) and quantified using Quantity One version 4.1.0 (Bio-Rad) according to the instructions. For data quantification, band intensity of each blot was calculated as ratio relative to β-actin. The intensity ratio of the control group was set as 100%, and the intensity of other treatment groups were expressed as percentage to the control group.

### Statistical analysis

Results were expressed as the mean ± SEM. Data were evaluated using one-way analysis of variance (ANOVA) for *post hoc* comparisons (SPSS 13.0). Data that passed the homogeneity test were analyzed by the one-way ANOVA Least Significant Difference (LSD) test. Data that did not pass the homogeneity test were analyzed by the one-way ANOVA Dunnett’s T3 test. In all cases, *p <* 0.05 was considered statistically significant.

## Abbreviations

ACC: Anterior cingulate cortex; HT: Hypothyroidism; sEPSC: Excitatory and inhibitory postsynaptic currents; sIPSC: Inhibitory postsynaptic currents; T3: Tri-iodothyronine; T4: Thyroxine.

## Competing interests

The authors declare that they have no competing interests.

## Authors’ contributions

YJ, JYZ, WZ and SW performed the experiments. YZF analyzed the data. KFD wrote the manuscript. All authors approved the final version of the manuscript.
